# Retinoic Acid Induces Apoptosis of Prostate Cancer DU145 Cells through Cdk5 Overactivation

**DOI:** 10.1155/2012/580736

**Published:** 2012-12-13

**Authors:** Mei-Chih Chen, Chih-Yang Huang, Shih-Lan Hsu, Eugene Lin, Chien-Te Ku, Ho Lin, Chuan-Mu Chen

**Affiliations:** ^1^Department of Life Sciences, National Chung Hsing University, Taichung 40227, Taiwan; ^2^Department of Health and Nutrition Biotechnology, Asia University, Taichung 41354, Taiwan; ^3^Graduate Institute of Basic Medical Science, China Medical University, Taichung 40402, Taiwan; ^4^Department of Education and Research, Taichung Veterans General Hospital, Taichung 40705, Taiwan; ^5^Department of Urology, Chang Bing Show Chwan Memorial Hospital, Changhua 50544, Taiwan; ^6^Agricultural Biotechnology Center, National Chung Hsing University, Taichung 40227, Taiwan; ^7^Graduate Institute of Rehabilitation Science, China Medical University, Taichung 40402, Taiwan; ^8^Department of Urology, University of Texas Southwestern Medical Center, Dallas, TX 75390, USA

## Abstract

Retinoic acid (RA) has been believed to be an anticancer drug for a long history. However, the molecular mechanisms of RA actions on cancer cells remain diverse. In this study, the dose-dependent inhibition of RA on DU145 cell proliferation was identified. Interestingly, RA treatment triggered p35 cleavage (p25 formation) and Cdk5 overactivation, and all could be blocked by Calpain inhibitor, Calpeptin (CP). Subsequently, RA-triggered DU145 apoptosis detected by sub-G1 phase accumulation and Annexin V staining could also be blocked by CP treatment. Furthermore, RA-triggered caspase 3 activation and following Cdk5 over-activation were destroyed by treatments of both CP and Cdk5 knockdown. In conclusion, we report a new mechanism in which RA could cause apoptosis of androgen-independent prostate cancer cells through p35 cleavage and Cdk5 over-activation. This finding may contribute to constructing a clearer image of RA function and bring RA as a valuable chemoprevention agent for prostate cancer patients.

## 1. Introduction

Prostate cancer is a common type of malignancy in men. It is known that androgen plays important roles in the formation and progression of prostate cancer [1]. In the early stage, surgery and androgen-depletion therapy can be useful. However, after a couple of years of androgen ablation therapy, the disease will reemerge and become androgen-independent. In addition, the stabilization of androgen receptor protein in prostate cancer cells changes with the transition of androgen requirement [2]. The fatal advanced prostate cancer will be aggressive and metastatic which cannot be effectively treated [3].

Cdk5 (cyclin-dependent kinase 5), a proline-directed serine/threonine kinase, belongs to Cdk (cyclin-dependent kinase) family based on the high level of sequence homology with the other Cdks [4]. However, the atypical member Cdk5 does not participate directly in cell cycle progression but plays important roles in the development of central nervous system and progression of neurodegenerative diseases [5]. Association with a noncyclin regulator, p35, is required for Cdk5 activation and therefore maintaining neuronal behaviors [6]. However, cleavage of p35 by a calcium-activated protease, Calpain, generates a truncated fragment p25 which overactivates Cdk5 and consequently causes neuronal apoptosis [7]. Our previous studies have illustrated the important roles of Cdk5 not only in neurodegeneration [8] but also in neuronal differentiation [9]. Additionally, a series of reports reveal the importance of Cdk5 in cancer cells [10–13]. The previous studies also show that Cdk5 may contribute to the regulation of proliferation in thyroid cancer [14] and prostate cancer [15]. In addition, our data also indicate that the formation of Cdk5/p35 complex is sensitive to drug treatments and causes apoptosis in cancer cells [16, 17]. Therefore, Cdk5/p35 might be considered as a potential diagnostic and therapeutic target for cancers in the near future.

Retinoic acid (RA), which is the most potent form of a trophic factor vitamin A (retinol), possesses the capacity to inhibit cancer cell survival or to induce apoptosis in a variety of cancers [18–22]. In human body, vitamin A converts into *all-trans* retinoic acid through two oxidation steps [23], and RA acts via both autocrine and paracrine signaling pathways [24]. In target cells, RA enters the nucleus and binds to RA receptor-retinoic X receptor (RAR-RXR) heterodimers and plays as a transcription factor to regulate the downstream genes and control physiological functions including vision, immune function, cell proliferation, and differentiation [24, 25].

Several lines of evidence indicate that retinoic acid may obstruct growth of cancer cells and even cause apoptosis [26–28]. Our previous results indicate that RA may cause apoptosis through p21 expression and cdc2 activation in human hepatoma Hep3B cells [20]. In cervical cancer cells, we also provide evidence indicating the involvement of Cdk5 in RA-induced apoptosis [17]. Retinoic acid and its derivatives have been reported to have the potential to treat prostate cancer in cell and animal models [29–31]. Although the clinical effect of retinoic acid alone is controversial [32, 33], combination of retinoids and other anticancer drugs acts synergistically to inhibit the growth of prostate cancer [34, 35]. Therefore, understanding how retinoic acid acts in prostate cancer cells is useful for developing new usage of retinoids in therapeutic trials. In this study, RA was found to induce cleavage of p35 into p25 and cause Cdk5 overactivation as well as subsequent apoptosis in human prostate cancer cell line DU145. This information might be helpful in therapeutic strategy or chemoprevention for prostate cancer patients.

## 2. Materials and Methods

### 2.1. Cell Culture and Transfection of siRNA

Human prostate cancer cell line, DU145 (BCRC no. 60348) was purchased from Bioresource Collection and Research Center, Taiwan. DU145 cells were cultured in MEM medium (Sigma, St. Louis, MO, USA) plus 10% fetal bovine serum (Gibco, Carlsbad, CA, USA), and penicillin/streptomycin (Sigma, St. Louis, MO, USA) at 37°C in a humidified atmosphere at 5% CO_2_. Cells were treated with different dosages (0.1, 1, 10 *μ*M) of *all-trans* retinoic acid (Sigma, St. Louis, MO, USA) individually in serum-free medium after serum starvation for 24 hrs. siRNA-*Cdk5* and nonspecific control of siRNA were purchased from Dharmacon (SMARTpool 60-097 and D-001206-13-05, Pittsburg, PA, USA). Introduction of siRNAs into cells was performed by using Lipofectamine 2000 (Invitrogen, Grand Island, NY, USA) with 50 pmol siRNA/10^5^ cells.

### 2.2. MTT Assay

The modified colorimetric 3-(4,5-dimethylthiazol-2-yl)-2,5-diphenyltetrazolium bromide (MTT) assay was performed to quantify the proliferation of DU145 cells. Yellow MTT compound (Sigma, St. Louis, MO, USA) is converted by living cells into blue formazan, which is soluble in DMSO. The blue staining was measured using an optical density reader (Athos-2001, Australia) at 570 nm [14].

### 2.3. BrdU Cell Proliferation Assay

Cell proliferation was analyzed based on the incorporation of BrdU (5-bromo-2′-deoxyuridine) into the synthesis DNA during cell proliferation. The BrdU assays were performed using a colourimetric cell proliferation BrdU-ELISA kits (Roche Applied Science, Mannheim, Germany) according to the manufacturer's instruction. 

### 2.4. Trypan Blue Staining Assay

Cells were stained with trypan blue as described previously [2]. In brief, cells were cultured in 24-well plates; after being collected by trypsinization and suspended in PBS with 0.2% trypan blue (Sigma, St. Louis, MO, USA), the viable cells were counted by hemocytometer.

### 2.5. Immunoprecipitation and Western Blotting Analysis

Cell lysate was produced in lysis buffer (20 mM Tris-HCl (pH 7.4), 1% NP-40, 137 mM NaCl, 50 *μ*M EDTA, protease inhibitor cocktail, and 1 mM PMSF) or extract buffer (100 mM NaCl, 1 mM EDTA, 1 mM EGTA, 1 mM NaF, 20 mM Na_4_P_2_O_7_, 2 mM Na_3_VO_4_, 1% Triton X-100, 10% Glycerol, 0.1% SDS, 0.5% deoxycholate, 1 mM PMSF, and protease inhibitor cocktail) for immunoprecipitation. Immunoprecipitates were collected by binding with ExactaCruz (Santa Cruz Biotechnology, Santa Cruz, CA, USA). Proteins were analyzed by direct Western blotting (30 *μ*g/lane) or blotting after immunoprecipitation (1-2 mg/immunoprecipitation). Application of antibodies included anti-Cdk5 antibody (05-364, Upstate Biotechnology, Lake Placid, NY, USA), anti-p35 antibody (sc-820, Santa Cruz Biotechnology, Santa Cruz, CA, USA), anti-actin antibody (MAB1501, Millipore, Temecula, CA, USA), anti-cleaved caspase 3 (no. 9661, Cell Signaling, Danvers, MA, USA), and peroxidase-conjugated anti-mouse, anti-rabbit, or anti-goat antibodies (Jackson ImmunoResearch Laboratory, West Grove, PA, USA). ECL detection reagent (Perkin Elmer, Boston, MA, USA) was used to visualize the immunoreactive proteins on PVDF membrane (Perkin Elmer, Boston, MA, USA).

### 2.6. *In Vitro* Cdk5 Kinase Assay

Kinase assay was performed by washing immunoprecipitates 3 times with kinase reaction buffer (50 mM HEPES (pH 7.0), 10 mM MgCl_2_, and 1 mM DTT). The ExactaCruz beads with target proteins were incubated in kinase reaction buffer containing 2 *μ*g of substrate (histone H1, 14-155, Upstate Biotechnology, Lake Placid, NY, USA), 10 *μ*L of magnesium/ATP mixture (Upstate Biotechnology, Lake Placid, NY, USA), 5 *μ*L 5X assay dilution buffer (100 mM MOPS (pH 7.2), 125 mM *β*-glycerophosphate, 25 mM EGTA, 5 mM Na_3_VO_4_, and 5 mM dithiothreitol), and 1–3 *μ*Ci of [^32^P]ATP in a final volume of 40 *μ*L at 30°C for 30 min. The strength of phospho-histone H1 was identified by 10% SDS-polyacrylamide gel electrophoresis and visualized on the X-ray film (Fujifilm, Japan).

### 2.7. Statistics

All values are given as the mean ± standard error of the mean (SEM). In all cases, means were examined by Student's *t*-test. A difference between two means was considered statistically significant when *P* < 0.05.

## 3. Results

### 3.1. Effects of Retinoic Acid on Proliferation of DU145 Cells

**Figure 1:**
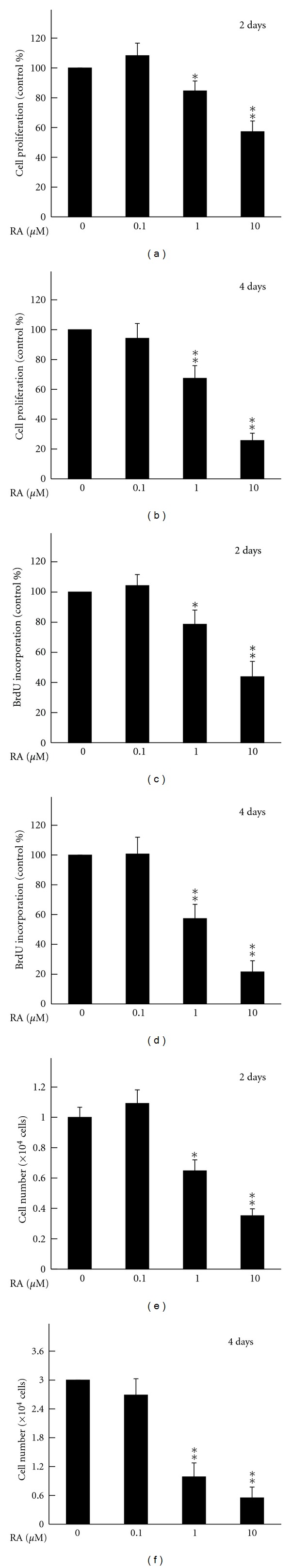
RA induces proliferation inhibition of DU145 cells. DU145 cells were treated with different dosages of RA after 1-day serum-free pretreatment. After 2-day or 4-day incubations, cells were performed (a, b) MTT assay, (c, d) BrdU assay, and (e, f) cell number counting as described in Materials and Methods (*n* = 6). Control value of cell proliferation was set at 100%. The values of error bars are given as the mean ± SEM. * and **, *P* < 0.05 and *P* < 0.01  versus the group of RA = 0.

*All-trans* retinoic acid (RA) was used to treat prostate cancer cell line, DU145, for 2 and 4 days. Cell proliferation was determined by MTT assay and BrdU incorporation as described in Materials and Methods. The results of MTT assay showed that RA could inhibit proliferation of DU145 cells in dose-dependent manners (Figures 1(a) and 1(b)) as well as BrdU cell proliferation assay (Figures 1(c) and 1(d)), in which, 1 *μ*M and 10 *μ*M RA significantly decreased DU145 proliferation after 2- or 4-day treatments. Additionally, cell number counting (by trypan blue staining) was performed to detect the viable cells after RA treatment. The data indicated that the cell numbers were dose-dependently decreased after application of RA which is parallel to the results of both MTT and BrdU assays (Figures 1(e) and 1(f)).

### 3.2. p35 Cleavage and Cdk5 Overactivation Are Triggered by RA Treatment

**Figure 2:**
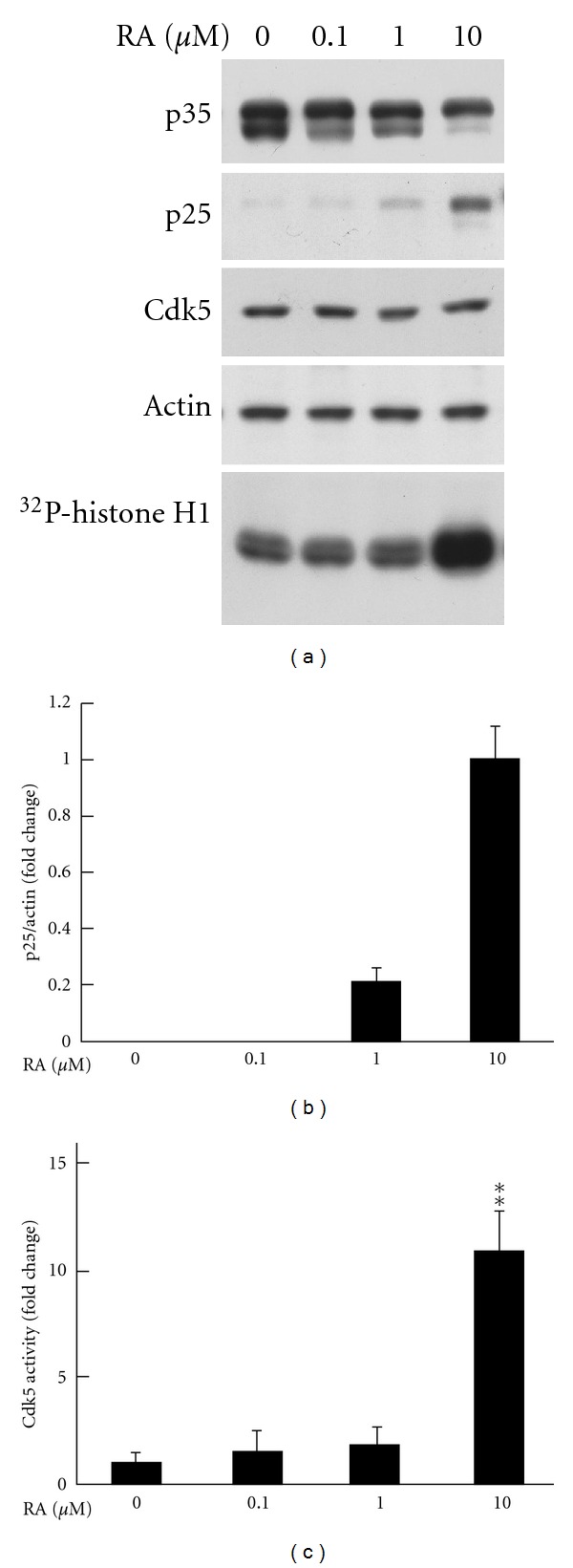
p35 cleavage and Cdk5 activation are dose-dependently triggered by RA. DU145 cells were treated with different dosages of RA after 1-day serum-free pretreatment. (a) After 4-day incubation, protein was extracted from cell lysate and p35/p25 and Cdk5 immunoblottings were performed as described in Materials and Methods. *β*-actin serves as an internal control. Cdk5 activity was detected by *in vitro* kinase assay using histone H1 as a substrate. (b, c) The quantitative results, respectively, revealed the effects of RA on p25 formation and Cdk5 activation. The independent experiments were repeated 3 times. Data are represented as means ± SEM; **P < 0.01 versus the group of RA = 0.

In order to investigate the conversion of p35 to p25 and the alteration of Cdk5 activity after RA treatment, different doses of RA were administered to DU145 culture medium for 4 days and the protein levels of p35, p25, and Cdk5 were detected by immunoblotting. Kinase assay was performed to detect Cdk5 activity as described in Materials and Methods. The data showed that increasing dosages of RA treatment gradually resulted in the decreases of p35 protein while p25 formation was oppositely increased (Figure 2(a)). The data of *in vitro* kinase assay showed that Cdk5 overactivation was accompanied with p25 formation. The quantified results of p25 protein levels and Cdk5 activity were, respectively, shown in Figures 2(b) and 2(c).

### 3.3. p35 Cleavage Inhibitor Neutralizes RA-Triggered Cdk5 Overactivation

**Figure 3:**
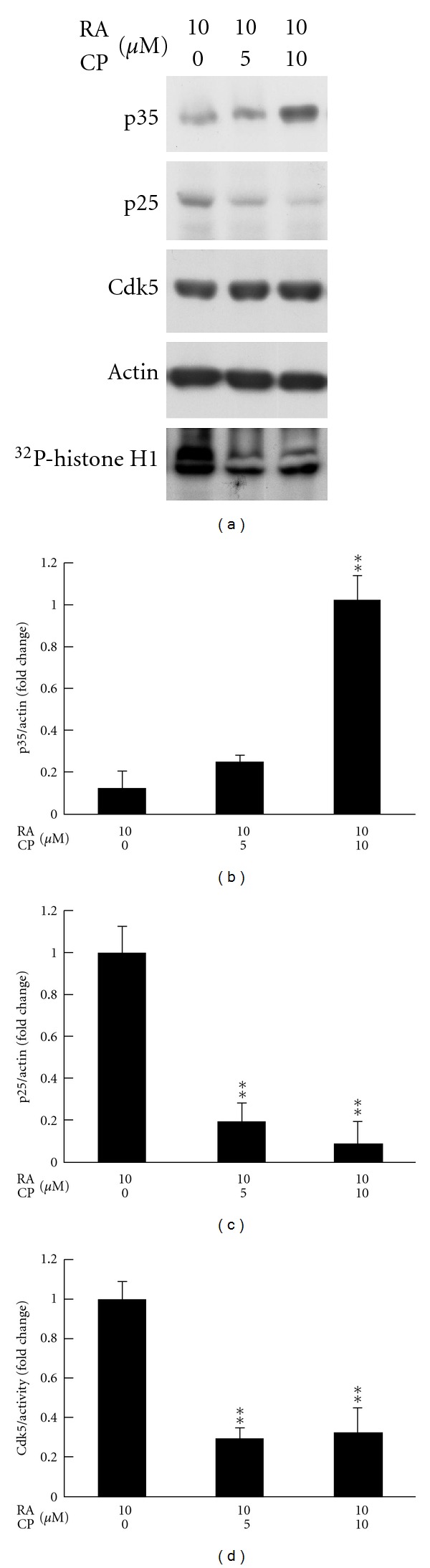
RA-triggered Cdk5 activation is blocked by p35 cleavage inhibitor. DU145 cells were treated with different dosages of Calpeptin (CP) plus 10 *μ*M RA after 1-day serum-free pretreatment. After 4-day incubation, protein was extracted from cell lysate and p35/p25 and Cdk5 immunoblottings were performed as described in Materials and Methods. *β*-actin serves as an internal control. Cdk5 activity was detected by *in vitro* kinase assay using histone H1 as a substrate. (b–d) The quantitative data respectively revealed the results of p35 cleavage, p25 formation, and Cdk5 activation. The independent experiments were repeated 3 times. Data are represented as means ± SEM; **P < 0.01 versus control group (CP = 0).

Calpeptin (CP) is a potent inhibitor of Calpain and has been used to block p35 cleavage in prostate cancer cells in previous study [16]. Here, we found that treatment of 10 *μ*M CP for 4 days effectively prevented RA-induced p25 formation and maintained p35 protein levels in DU145 cells (Figure 3(a)). In addition, CP administration also successfully prevented Cdk5 over-activation triggered by RA while Cdk5 protein levels were not affected. The quantified results of p35/p25 protein levels and Cdk5 activity were, respectively, shown in Figures 3(b)–3(d). These results suggest that RA treatment might trigger Cdk5 overactivation through p35 cleavage.

### 3.4. p35 Cleavage Inhibitor Obstructs RA-Triggered DU145 Apoptosis

**Figure 4:**
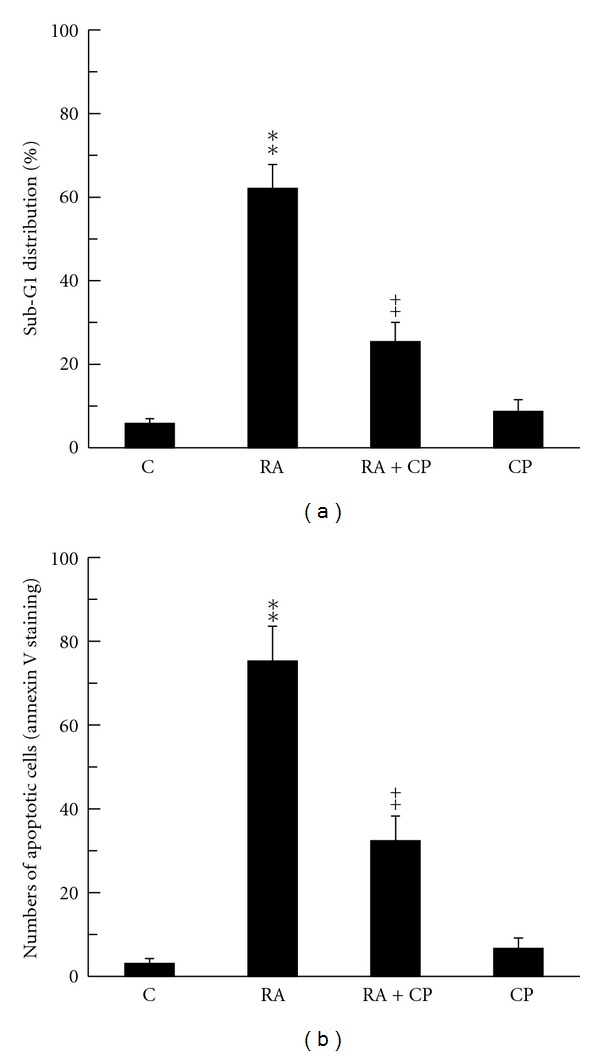
Treatment of p35 cleavage inhibitor blocks RA-induced DU145 apoptosis. DU145 cells were treated as follows: control, RA (10 *μ*M), RA + CP, CP (10 *μ*M) for 4 days after 1-day serum-free pretreatment. Cell apoptosis was analyzed by sub-G1 distribution (a) and Annexin V staining (b) as described in Materials and Methods. Sub-G1 distribution was detected by flow cytometry with propidium iodide staining. Annexin V staining was performed by specific antibody with FITC conjugation. The apoptotic cells were counted per 100 cells in a random microscopic field. The independent experiments were repeated 3 times. Data are represented as means ± SEM; **P < 0.01 versus control group; ^++^ *P* < 0.01 versus RA group.

The previous study indicates that p25 formation may lead to apoptosis of prostate cancer cells [16]. Therefore, whether RA is able to induce DU145 apoptosis through p25 formation is interesting to investigate. DU145 cells were treated with 10 *μ*M RA in the presence or absence of p35 cleavage inhibitor CP for 4 days. The apoptotic cells were evaluated by sub-G1 phase accumulation (DNA fragmentation) and Annexin V staining. By analyzing sub G1 phase accumulation using flow cytometry, RA significantly triggered DU145 apoptosis, and in contrast, CP effectively prevented the apoptotic effect of RA (Figure 4(a)). Besides, DU145 cells were stained by Annexin V antibody conjugated with FITC after 4-day treatments of RA in the presence or absence of CP, and the FITC-positive cells were randomly counted per 100 cells through fluorescent microscope. Similar to the results of sub G1 accumulation, inhibiting p25 formation by CP effectively prevented RA-triggered DU145 apoptosis (Figure 4(b)). In which, CP treatment alone did not affect DU145 apoptosis.

### 3.5. Inhibition of p25 Formation Declines Caspase 3 Activation

**Figure 5:**
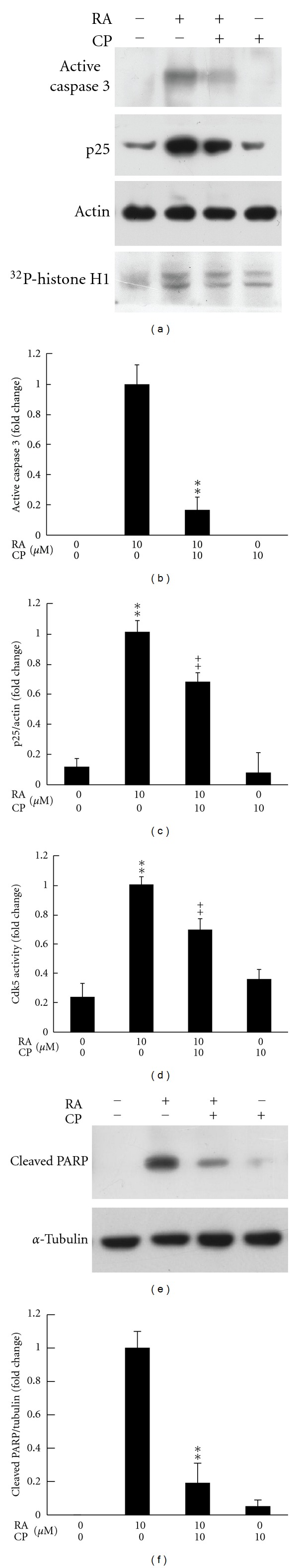
RA-triggered caspase 3 activation can be diminished by p35 cleavage inhibitor. DU145 cells were treated as follows: control, RA (10 *μ*M), RA + CP, CP (10 *μ*M) for 4 days after 1-day serum-free pretreatment. (a) Cleaved caspase 3, p25, and (e) cleaved PARP were detected by immunoblotting with specific antibodies as described in Materials and Methods. *β*-actin and *α*-tubulin serve as internal controls. Cdk5 activity was detected by *in vitro* kinase assay using histone H1 as a substrate. (b–d) and (f) The quantitative data, respectively, revealed the results of cleaved caspase 3, p25 formation, Cdk5 activation, and cleaved PARP. The independent experiments were repeated 3 times. Data are represented as means ± SEM; **P < 0.01 versus control group; ^++^ *P* < 0.01 versus RA alone group.

In addition to using sub-G1 accumulation and Annexin V staining, caspase 3 activation (cleavage) and PARP cleavage which is catalyzed by caspase 3 were then monitored by immunoblotting as another apoptotic indication. Consistent to the results in Figure 4, CP treatment did diminish RA-triggered activation of caspase 3 as well as the cleavage of PARP which were detected by specific antibody (Figures 5(a) and 5(e)). Moreover, the activations of Cdk5 and caspase 3 were sequentially induced and parallel with the protein levels of p25 after CP administration. The quantified results of activated caspase 3, p25 protein levels, PARP cleavage, and Cdk5 activity were, respectively, shown in Figures 5(b)–5(d) and 5(f). These results strongly suggest the correlation between RA-triggered p25-dependent Cdk5 over-activation and DU145 apoptosis.

### 3.6. Cdk5 Knockdown Blocks RA-Triggered DU145 Apoptosis

**Figure 6:**
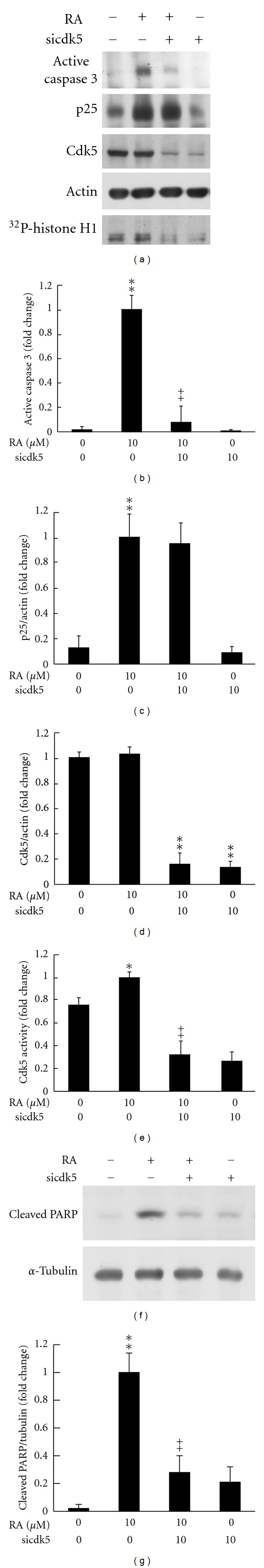
RA-triggered caspase 3 activation can be blocked by Cdk5 knockdown. DU145 cells were treated as follows: control, RA (10 *μ*M), RA + sicdk5, sicdk5 (5 pmol/10^4^ cells) for 4 days after 1-day serum-free pretreatment. The control group was transfected with siRNA-control. (a, f) Cleaved caspase 3, p25, Cdk5, and cleaved PARP proteins were detected by immunoblotting with specific antibodies as described in Materials and Methods. *β*-actin serves as an internal control. Cdk5 activity was detected by *in vitro* kinase assay using histone H1 as a substrate. ((b)–(e), (g)) The quantitative data, respectively, revealed the results of cleaved caspase 3, p25 formation, Cdk5, cleaved PARP protein levels, and Cdk5 activation. The independent experiments were repeated 3 times. Data are represented as means ± SEM; * and **P < 0.05 and *P* < 0.01 versus control group; ^++^ *P* < 0.01 versus RA = 10 group.

Since Cdk5 activity might be sensitive to RA-triggered apoptosis, we next performed Cdk5 knockdown to verify the necessity of Cdk5 in RA-induced DU145 apoptosis. Although p25 formation was not affected, the activation of Cdk5 was declined by Cdk5 knockdown and the activation of caspase 3 was also diminished (comparing lanes 2 and 3 in Figure 6(a)). The quantified results of caspase 3 activation, p25, Cdk5 protein levels, and Cdk5 activity were, respectively, shown in Figures 6(b)–6(e). In addition to the activation of caspase 3, PARP cleavage was also declined while Cdk5 was knockdown (Figure 6(f)).  Figure 6(g) showed the quantified results of PARP cleavage. Taken together, these results, again, suggest that RA triggered DU145 apoptosis through p35 cleavage and subsequent Cdk5 over-activation.

## 4. Discussion

Cdk5 has versatile biological functions in cells discovered in recent decades [5, 13]. Our previous results and other reports both suggest that physiological regulation/activation of Cdk5 contributes to the maintenance of cancer cell growth [10, 11, 14]. Specifically, we found that protein stability of androgen receptor is regulated by physiological activation of Cdk5 in prostate cancer cells and cell proliferation is, therefore, promoted [15]. As regards androgen stimulation, our previous report indicates the role of Cdk5 in androgen production in male testis [36], which implies that Cdk5 regulation of systemic androgen secretion may contribute to androgen-dependent prostate cancer growth. On the other hand, we found that over-activation of Cdk5 by the truncated p25 generated from p35 cleavage is critical to drug-triggered apoptosis of cancer cells [16, 17]. In this study, we demonstrate the mechanism that how RA triggers apoptosis of androgen-independent prostate cancer cells, in which, we emphasize the importance of p25/Cdk5 deregulation in RA-triggered apoptosis in DU145 cells. These observations suggest that under physiological management, Cdk5 activation is modulated by p35 expression which maintains the activity of Cdk5 under a regular level. Once a stress happens (such as drug treatment, hypoxia, or immune response), p35 is spitted into p25 which excessively activates Cdk5 and leads to cell apoptosis [16, 17]. Therefore, we hypothesize that Cdk5 might modulate both proliferation and apoptosis of prostate cancer cells and the activation status of Cdk5 might determine the different cell fates.

RA is believed to be an important factor in the development of central nervous system and induces neuronal differentiation in embryonal carcinoma cells [37]. Some studies have revealed the connection between RA and Cdk5 in nervous system. RA treatment activates Cdk5-mediated PI3K/Akt and ERK pathways which cause Bcl-2 upregulation and prevent apoptosis during neuronal differentiation [38]. Expressions of Cdk5 and its regulators as well as its activity are increased during RA-induced neuronal differentiation in NT2 cells [39]. Moreover, in human neuroblastoma SK-N-BE(2)C cells, RA treatment increases the expressions of Cdk5 and p35 through ERK and cAMP-dependent protein kinase A pathway [40]. These lines of evidence suggest that Cdk5 might involve RA-related effects in cancer cells, such as differentiation and apoptosis. 

Under androgen-dependent stage, treatments for prostate cancer are more effective. However, once it progresses into androgen-independent state, there are rare efficient strategies for treatment. Based on the above, this study focused on the anticancer effect of RA on androgen-independent prostate cancer cells. The administration of RA inhibited the proliferation and viability of DU145 cells which is an androgen-independent cell line. A series of analyses were taken to confirm that the inhibition of cell growth is consequent to apoptosis induced by RA treatment. In addition to DU145 cells, RA administration also induced caspase 3 activation as well as p25 formation in PC3 cells (see Supplementary Figure  1 of the Supplementary Material available online at doi:10.1155/2012/580736). In order to silence the interference from serum albumin and other growth factors in serum [41], cells were starved for 24 hr and treated with RA in serum-free medium. Besides, 9-*cis* RA and 13-*cis* RA showed slight effects on p25 formation and caspase 3 activation compared to *all-trans* RA (Supplementary Figure  2). 

Calcium ion is a second messenger and participates in the processes of cell activation [42]. Intracellular calcium elevation is important for apoptosis and regulates several key steps in the apoptotic pathway [43]. In leukemic cells, retinoic-acid-related compound had been proved to cause cell apoptosis through calcium modulation [44]. This evidence raises the possibility that retinoids might attack cancer cells through calcium control. In our study, Calpeptin, an inhibitor of Calpain which is sensitive to intracellular calcium, was used to inhibit the formation of p25, and therefore the apoptotic effects of RA in DU145 cells were restricted. To verify the involvement of calcium in RA actions, EGTA was used as a calcium chelator in DU145 as described in previous study [16] and was found that it could block RA-triggered p25 formation and caspase-3 activation (Supplementary Figure  3). According to these results, we suggest that RA might alter the calcium homeostasis which triggers Calpain-dependent p35 cleavage and consequently causes apoptosis in DU145 cells.

Several reports indicate that the metabolism of retinol conversion and its receptors are impaired in several types of cancer including prostate cancer [45]. The expression patterns of RA receptor subtypes in prostate cancer cells may influence the action of RA [46]. Variant effects of RA on ERK1/2 phosphorylation and cAMP accumulation were observed in normal and malignant human prostate epithelial cells [47]. This suggests that retinoids might be unfavorable to tumorigenesis. Based on this point, RA administration has been applied to cancer treatment and may induce growth inhibition and apoptosis of prostate cancer cells [48–50]. Furthermore, combinations of RA and other cancer therapeutic agents synergistically induce apoptosis of prostate cancer cells, which suggests that RA might become a helper to current cancer treatments [51, 52]. However, the molecular mechanisms by which RA affects the fate of prostate cancer cells are still unclear. Here, we report that RA might cause apoptosis of androgen-independent prostate cancer cells through novel pathway, in which p35 cleavage and Cdk5 over-activation were involved. Blockade of Cdk5 over-activation could successfully obstruct RA-induced cell apoptosis. RA itself is a nutrient necessary for life-sustaining and also a pharmacological agent for decades [53]. Here, we consider RA as an anti-cancer agent, in which we believe that our evidence contributes to the literature by elucidating the mechanisms of RA-induced apoptosis of androgen-independent prostate cancer cells. This finding might be applied to the therapeutic stratagem through AR administration and combination with other agents for prevention of processing malignance of prostate cancer.

## Supplementary Material

Supplementary Figure 1: RA triggered p25 formation and caspase-3 activation in prostate PC3 cancer cells. PC3 cells were treated as follows: control, RA (10 **μ**M), RA*+*CP, CP (10 **μ**M) for 4 days after 1-day serum-free pretreatment. Cleaved caspase-3 and p25 were detected by immunoblotting with specific antibodies as described in Materials and Methods. **β**-actin served as an internal control.
Supplementary Figure 2: The comparison of effects of *all*-*trans*-retinoic acid, 9-*cis*-retinoic acid, and 13-*cis*-retinoic acid on p25 formation and caspase-3 activation in prostate DU145 cancer cells. DU145 cells were treated as follows: control, *all*-*trans*-retinoic acid (10 **μ**M), 9-*cis*-retinoic acid (10 **μ**M), or 13-*cis*-retinoic acid (10 **μ**M) for 4 days after 1-day serum-free pretreatment. Cleaved caspase-3 and p25 were detected by immunoblotting with specific antibodies as described in Materials and Methods. **β**-actin served as an internal control.
Supplementary Figure 3: RA-triggered p25 formation and caspase-3 activation can be diminished by calcium chelator, EGTA, in DU145 cells. DU145 cells were treated as follows: control, RA (10 **μ**M), RA*+*EGTA, EGTA (0.5 mM) for 4 days after 1-day serum-free pretreatment. Cleaved caspase-3 and p25 were detected by immunoblotting with specific antibodies as described in Materials and Methods. **β**-actin served as an internal control.

## Abbreviations

Cdk5: Cyclin-dependent kinase 5
RA: Retinoic acid
CP: Calpeptin.
