# Supplementary material for: Retinoic Acid Induces Apoptosis of Prostate Cancer DU145 Cells through Cdk5 Overactivation
Source: Evid Based Complement Alternat Med. 2012 Dec 13;2012:580736. doi: 10.1155/2012/580736 (PMC3532922; doi:10.1155/2012/580736)
Supplement: Supplementary file 1 — Supplementary Figure 1: RA triggered p25 formation and caspase-3 activation in prostate PC3 cancer cells. PC3 cells were treated as follows: control, RA (10 μM), RA+CP, CP (10 μM) for 4 days after 1-day serum-free pretreatment. Cleaved caspase-3 and p25 were detected by immunoblotting with specific antibodies as described in Materials and Methods. β-actin served as an internal control. Supplementary Figure 2: The comparison of effects of all-trans-retinoic acid, 9-cis-retinoic acid, and 13-cis-retinoic acid on p25 formation and caspase-3 activation in prostate DU145 cancer cells. DU145 cells were treated as follows: control, all-trans-retinoic acid (10 μM), 9-cis-retinoic acid (10 μM), or 13-cis-retinoic acid (10 μM) for 4 days after 1-day serum-free pretreatment. Cleaved caspase-3 and p25 were detected by immunoblotting with specific antibodies as described in Materials and Methods. β-actin served as an internal control. Supplementary Figure 3: RA-triggered p25 formation and caspase-3 activation can be diminished by calcium chelator, EGTA, in DU145 cells. DU145 cells were treated as follows: control, RA (10 μM), RA+EGTA, EGTA (0.5 mM) for 4 days after 1-day serum-free pretreatment. Cleaved caspase-3 and p25 were detected by immunoblotting with specific antibodies as described in Materials and Methods. β-actin served as an internal control. [file 580736.f1.pdf]

Supplementary Figure 1

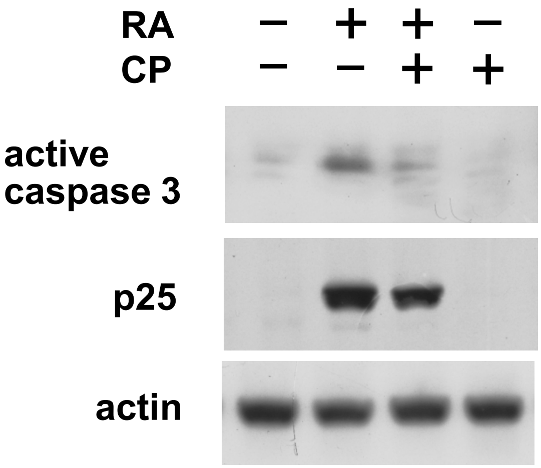

RA also triggered p25 formation and caspase-3 activation in prostate PC3 cancer cells. PC3 cells were treated as follows: control, RA (10 μM), RA+CP, CP (10 μM) for 4 days after 1-day serum-free pretreatment. Cleaved caspase-3 and p25 were detected by immunoblotting with specific antibodies as described in Materials and Methods. β-actin served as an internal control.

Supplementary Figure 2

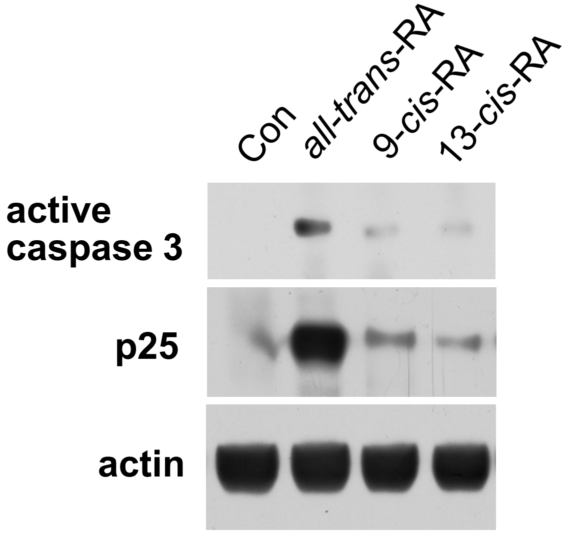

The comparison of effects of *all-trans*-retinoic acid, 9-*cis*-retinoic acid, and 13-*cis*-retinoic acid on p25 formation and caspase-3 activation in prostate DU145 cancer cells. DU145 cells were treated as follows: control, *all-trans*-retinoic acid (10 μM), 9-*cis*-retinoic acid (10 μM), or 13-*cis*-retinoic acid (10 μM) for 4 days after 1-day serum-free pretreatment. Cleaved caspase-3 and p25 were detected by immunoblotting with specific antibodies as described in Materials and Methods. β-actin served as an internal control.

Supplementary Figure 3

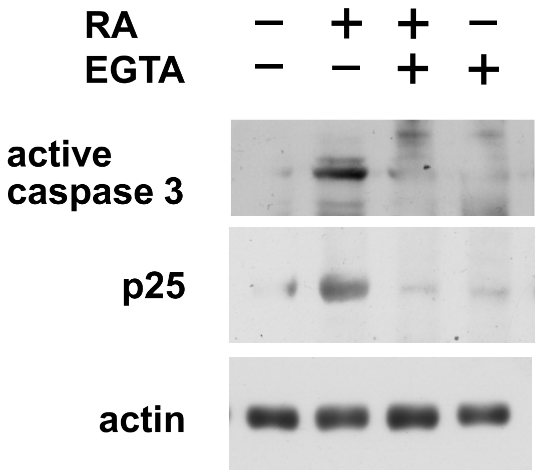

RA-triggered p25 formation and caspase-3 activation can be diminished by calcium chelator, EGTA, in DU145 cells. DU145 cells were treated as follows: control, RA (10 μM), RA+EGTA, EGTA (0.5 mM) for 4 days after 1-day serum-free pretreatment. Cleaved caspase-3 and p25 were detected by immunoblotting with specific antibodies as described in Materials and Methods. β-actin served as an internal control.
